# Male starling floaters preferentially visit nests of males with reduced resource holding potential

**DOI:** 10.1098/rsbl.2023.0376

**Published:** 2024-03-06

**Authors:** Eduardo Gómez-Llanos, Iraida Redondo, Lorenzo Pérez-Rodríguez, Diego Gil

**Affiliations:** ^1^ Departamento de Ecología Evolutiva, Museo Nacional de Ciencias Naturales (CSIC), José Gutiérrez Abascal 2, 28006 Madrid, Spain; ^2^ Instituto de Investigación en Recursos Cinegéticos (IREC), CSIC-UCLM-JCCM, Ronda de Toledo 12, 13005 Ciudad Real, Spain

**Keywords:** floaters, intraspecific competition, RFID identification, resource holding potential, sexual selection

## Abstract

Floaters are sexually mature individuals that are not able to reproduce by defending breeding resources. Floaters often visit active nests, probably to gather public information or to compete for a nesting site. We tested the hypothesis that floaters preferentially prospect nests in which they have a better chance of taking over, and that they do so by assessing the owners' resource holding potential (RHP). We manipulated the flight capacity of male and female breeders in a population of spotless starlings (*Sturnus unicolor*) by clipping two flight feathers per wing before egg laying, thus increasing their wing-load and likely impairing their condition. We subsequently monitored breeder and floater activity by means of transponder readers during the nestling period. We found that nests owned by wing-clipped males were visited by a greater number of male floaters than control nests. This effect was absent in the case of wing-clipped females. The number of male floaters also increased with increasing nestling age and number of parental visits. The experiment shows that male floaters preferentially prospect nests in which the owner shows a reduced RHP, a strategy that likely allows them to evict weak owners and take over their nests for future reproductive attempts.

## 1. Introduction

Examining the interactions between breeding and non-breeding individuals is essential for understanding animal social dynamics and reproductive patterns. Studies investigating the behaviour of non-breeding individuals are particularly relevant to clarify the establishment and maintenance of social hierarchies, the rules of access to resources and the dynamics of mating strategies in animal populations [1].

In different groups of animals, the capacity to acquire and defend a breeding territory is key for reproduction and is known as *resource holding potential* (RHP) [2]. Floaters are sexually mature individuals in a population that are not able to reproduce due to their lower status or ability to obtain a territory [1,3,4], and some studies suggest that these individuals have a lower RHP [5,6] and are forced to wait for a breeding opportunity [7].

In cavity-nesting birds, securing a nest site is an essential requisite for breeding [8]. The availability of cavities is usually limited, leading to intense competition. In several species, non-breeding individuals often visit active nests during the breeding season [9–11]. Many studies refer to this behaviour as ‘prospecting’, assuming that these visits allow floaters to gather information about the nesting conditions or resources of breeding pairs [11–13]. This strategy is useful when there is important variation in habitat quality, since nestling condition could act as a proxy of the amount of resources in the area [13]. Another possibility is that floaters use these nest visits to gain fitness benefits via extra-pair paternity or conspecific brood parasitism [14]. Alternatively, in particular after egg laying, prospecting behaviour may be used to evaluate competitor fitness and guide overtaking attempts [7,15]. In some species, such as the spotless starling, nest sabotage and take-overs by both sexes are frequent events [16], often involving fights that can lead to the death of one of the birds (electronic supplementary material, figure S1). In addition, nests of secondary females of polygynous males are often poorly defended and become an easy target for floaters of both sexes [17].

If floaters use nest visits to gather information on breeder quality, we would expect that nests defended by poor quality individuals should be preferentially visited over those defended by high-quality birds. To test this hypothesis, we conducted a wing-clipping manipulation (as in [18]) in male and female breeders. This manipulation increases wing-load, and has been shown to result in a decrease in female body condition [18,19]. We assume that it has a similar effect in males, decreasing the RHP of both sexes. We expected that floaters would increase nest prospecting in the nests of manipulated birds. We predicted a sex-specific effect, wing-clipped individuals attracting a higher number of floaters of their own sex, as well as an increased number of visits per floater.

The study was performed in a long-term monitored population of spotless starlings (*Sturnus unicolor*), a highly social species that breeds in secondary cavities (i.e. tree holes made by other species). Both sexes are sexually mature from their first year of age, although, due to high competition for a nesting site, most males start breeding in their second or third year, whereas females do so in their first or second year [6]. This situation leads to a scenario with a large number of floaters (*ca* twice the breeding population in the case of males, as many as the breeding population in the case of females; E Gómez-Llanos, I Redondo, L Pérez-Rodríguez, D Gil 2018–2020, unpublished data), which is highly suitable to study floater's behavioural strategies.

## 2. Methods

The study population consists of 250 nest-boxes on an open woodland at Soto del Real (Madrid, Spain), which is fully occupied every year. Nest density is 3.72 boxes Ha^−1^, and the average distance to the nearest box is 22.1 m (s.d. = 8.7). The experiment took place in March 2020. Approximately one month before laying, we captured and marked adults with metal rings and subcutaneous passive integrated transponders (PIT tag) which can be detected by radiofrequency identification (RFID). We captured birds in the early morning, either by shutting the nests before the birds leave or with spring traps inside the box. By the end of March, most breeding adults (greater than 90%) were equipped with PIT tags, as well as a large number of floaters captured in boxes while prospecting, or that were PIT tagged as nestlings in the previous 2 years (1176 nestlings in 2018, and 1191 in 2019).

We randomly assigned nests to only one of the two wing-clipping treatments (male or female) or the control group, balancing treatments for males and females. The treatment consisted on clipping the fourth and fifth primary feathers of each wing at their base with a nail clipper [18]. Birds in the control group were similarly handled, but their plumage was left intact. Captures took place on average 30.3 days (s.d. = 9.6) before egg laying. We previously reported that, after controlling for pre-treatment body weight, wing-clipped females were 3.1% lighter than controls while brooding (males could not be caught at this time, but we assume that a similar effect would occur) [19]. The initial sample size was 24 control nests, 19 nests with wing-clipped females and 25 with wing-clipped males. In a given nest, we only manipulated one member of the pair. Some of the broods suffered sabotages or predation before the nestling stage, and some readers failed to work, so our final sample size for visiting data was reduced to 15 control pairs, 12 wing-clipped females and 17 wing-clipped males.

After the manipulation, we followed the nests to determine hatching, and used RFID technology to record parents and floater visits. We used readers (Trovan LID650) as described by Redondo *et al*. [6], that detect PIT tags when the bird fully introduces its neck inside the box. Since floaters tend to show higher prospecting activity during the nestling period than in incubation [11], we monitored nests during the peak of the nestling stage. Between ages 10 and 14, we set up the readers to work from 07.00 to 20.00. However, due to battery failure, the number of recording days and times varied slightly: mean = 4.4 days per nest (s.d. = 0.8), 10.3 h per day (s.d. = 3.4), 38.01 h in total per nest (s.d. = 8.9).

For each day and nest we calculated the number of different individuals and the total number of visits per nest. A floater was defined as any individual not breeding during the time covered by the readings (this included mostly year-round floaters, but also a few birds that had lost their brood and floaters that became breeders in second broods, after the experiment finished). We also calculated the provisioning rates of both parents. We used an 8 s cut-off filter between consecutive readings of the same bird to score provisioning rate or number of visits (we validated this approach using video-cameras: Gómez-Llanos *et al*. [20]). Since we do not know how long floaters spend per visit, our estimate of the number of visits can be taken to imply visiting time. Due to differences in reader or battery operation times, we calculated recording time for each nest and day (the time elapsed between the first and the last detection of any bird) and controlled for this measurement in all analyses.

### (a) Data analysis

All analyses were performed in R language v. 4.2.2 [21]. We evaluated if treatment (control, wing-clipped female or wing-clipped male) influenced the number of female and male floaters (separately) that visited each nest as well as the number of visits made by female and male floaters per nest. We also included as scaled covariates: (1) the amount of time that the readers worked in each nest, since the number of detections is expected to linearly increase with recording time; (2) the summed number of parental visits, as we expect nests with higher parental visiting rates to be more detectable; and (3) brood size and age, as nests are more easily detected when nestlings perform parent-absent begging with increasing age [22].

We used the package *glmmTMB* [23] to build generalized linear mixed models (GLMM). We explored different possible response distributions (Poisson, truncated Poisson, and negative binomial 1 and 2), and also included hurdle and zero-inflated structures. We selected the model with the lowest AIC value and an optimal *DHARMa* [24] fit. Models for the number of visiting male and female floaters were fitted using a Poisson distribution, whereas those on the number of visits were fitted using a hurdle model with a zero-inflated negative binomial 2 distribution, after subtracting one to each datum. All models included nest identity as random effect. Models on the number of visits also included floater identity as a random effect. Normality and homocedasticity were checked by inspecting model residuals using *DHARMa* [24], and marginal means were calculated with *emmeans* [25].

## 3. Results

### (a) Number of visitors

**Figure 1.**
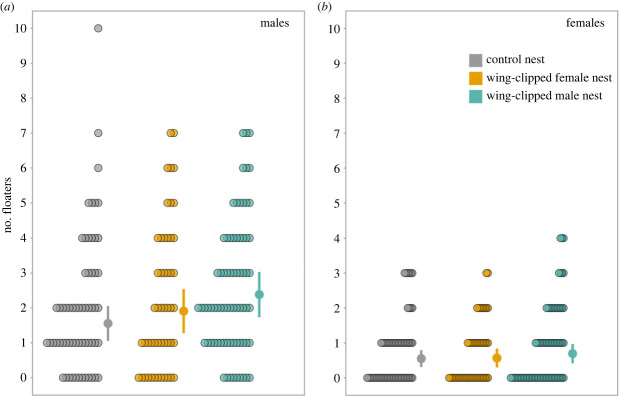
Number of male (*a*) and female (*b*) floaters per day prospecting nest boxes in relation to treatment and corrected by recording duration. Graphs include actual data points, marginal means and the 95% confidence interval per group, derived from GLMM Poisson models. Table 1 and text for detailed statistics and model details.

The number of male floaters was higher in those nest-boxes where the male owner had been wing-clipped than in control nests (table 1 and figure 1). This effect corresponds to an increase in 50% of the number of visitors per day (marginal means (SE) in the original scale: control versus male wing-clipped: 1.56 (0.3) versus 2.38 (0.3)). The number of male floaters increased with recording time and number of visits by parents and increased with nestling age (table 1). Larger brood sizes were not more likely to be visited.

**Table 1.** Effects of treatment, age, recording time, brood size and parental visits on the number of male (left) and female (right) floaters visitors to conspecific's nests. Results are from Poisson GLMMs. Figures for the random effect represent the variance plus the standard deviation in parenthesis. For the fixed effects, figures represent the estimate and the 95% confidence interval.

	males	females
random effects		
nest ID	0.20 (0.45)	0.27 (0.52)
fixed effects		
intercept	0.44 (0.16)**	−0.52 (0.21)*
wing-clipped female	0.20 (0.22)	0.01 (0.30)
wing-clipped male	0.43 (0.21)*	0.19 (0.28)
nestling age	0.12 (0.05)*	0.05 (0.09)
recording time	0.21 (0.09)*	0.41 (0.15)**
brood size	0.02 (0.08)	−0.05 (0.12)
parental visits	0.29 (0.09)**	0.18 (0.13)

**p < 0.01, *p < 0.05.

In the case of female floaters, we did not find differences in the number of birds visiting nest boxes in relation to treatment (table 1). As in males, the length of the recording period had a positive influence on the number of female floaters (table 1).

### (b) Number of visits

The number of visits made by male or female floaters was not affected by the wing-clipping treatment, nor could it be predicted by parental visits, nestling age or recording duration (table 2; electronic supplementary material, figure S2).

**Table 2.** Effects of treatment, age, recording time, brood size and parental visits on the number of visits made by male (left) and female (right) floaters to conspecific's nests. Results are from hurdle GLMM models with negative binomial 2 response distributions. See methods for statistical details. Figures as per table 1.

	males		females	
	zero-inflation part	conditional part	zero-inflation part	conditional part
random effects				
nest ID	0.004 (0.06)	0.008 (0.09)	1.5×10^-4^ (0.01)	0.121 (0.35)
floater ID (intercept)	0.001 (0.01)	0.033 (0.18)	3.2×10^-4^ (0.18)	0.755 (0.61)
fixed effects				
intercept	−14.50 (126.5)	1.253 (0.10)***	−12.97 (117.1)	0.93 (0.24)***
wing-clipped female	−0.438 (209.6)	−0.02 (0.14)	−2.25 (207.8)	−0.11 (0.33)
wing-clipped male	0.05 (162.5)	−0.13 (0.13)	−2.18 (127.7)	−0.07 (0.27)
nestling age	0.02 (71.7)	−0.003 (0.05)	0.85 (65.6)	−0.17 (0.11)
recording time	−0.02 (71.4)	0.02 (0.05)	1.08 (163.4)	0.01 (0.11)
brood size	0.02 (71.9)	−0.08 (0.06)	−0.73 (52.1)	0.11 (0.13)
parental visits	0.01 (71.19)	0.06 (0.05)	0.91 (60.9)	−0.04 (0.10)

***p < 0.001.

### (c) Brood success

Nests in this population show relatively high levels of failure due to sabotage, predation or desertion. In this study, 29.1% of control broods failed to fledge offspring. This percentage was not different between treatments (GLM, binomial link, *Χ*^2^ = 2.63, *p* = 0.268). Although the failure rate was lower for the male (16%) than for the female treatment (36%), there was no difference between groups (Tukey's *post hoc* tests, all *p* > 0.26).

## 4. Discussion

Our knowledge of territorial dynamics in bird species is biased towards the behaviour of owners, whereas floaters, which are key actors in territory conflicts, are typically neglected. By experimentally reducing the flight performance of breeders of both sexes [19], we tested if male and female floaters preferentially prospected nests owned by manipulated breeders. We found that this was the case for male birds, since nests in which the male owner had been wing-clipped attracted a larger number of male floaters than control nests. To the extent that this manipulation likely affected male RHP in the same way that it affected females', this pattern reinforces the idea that male floaters do not prospect nests randomly but favour those in which the male owner has a reduced RHP [7,15]. The intrusion of floaters in foreign nests often results in fights between the owner and the floater [11], suggesting that prospecting can result in nest takeovers, although no differences were found in our study in this respect.

Our experiment suggests that floaters are capable of assessing the condition and flight performance of the owners they visit and preferentially visit nests belonging to individuals with lower RHP, which could increase their chances of taking over a nest. Wing-clipping has been shown to reduce wing lift and increase flying costs [26,27], likely reducing the capacity to defend nests against intruders.

What external cues could floaters use to detect the reduced RHP of experimental birds? We found that parental provisioning was a positive predictor of number of male visitors, as found in other studies [11,12,28], suggesting that prospecting birds observe owners, and that nests with many visits are more easily located. However, the additional effect of male treatment on number of visitors suggests that the manipulation may have led to differences in the way that birds fly, probably reminiscent of a bird showing damaged plumage or moulting at the wrong time of year, possibly indicating lower RHP.

In contrast to males, we found no effect of the female treatment on female floater visits. We expected the same pattern as in males because female ownership can also change between first and second broods. However, there were fewer female than male floaters (164 versus 504 individuals in the study year), possibly implying a smaller statistical power to detect an effect. Alternatively, females may preferentially prospect during the egg-laying phase, when frequent conspecific brood parasitism and nest sabotages occur [14].

In conclusion, our results suggest that male floaters do not prospect random nests but preferentially visit nests with owners who are more likely to be evicted [7]. Our results are consistent with the hypothesis that floaters actively prospect nests taking into account their chances of future settlement [7,15].

## Data Availability

Research data supporting this work are available from the Figshare repository: https://figshare.com/s/6469a2756810f9770dd3 [29]. Supplementary material is available online [30].
